# Bioinformatics analysis identifies GLUD1 as a prognostic indicator for clear cell renal cell carcinoma

**DOI:** 10.1186/s40001-024-01649-2

**Published:** 2024-01-20

**Authors:** Shuang Liu

**Affiliations:** Department of Clinical Laboratory, Institute of Translational Medicine, Renmin Hospital of Wuhan University, Wuhan, 430060 Hubei People’s Republic of China

**Keywords:** GLUD1, Renal cell carcinoma, TCGA, Bioinformatics

## Abstract

**Background:**

Renal cell carcinoma (RCC) is a common primary tumor of the kidney and is divided into three major subtypes, of which clear cell renal cell carcinoma (ccRCC) has the highest incidence. Glutamate dehydrogenase 1 (GLUD1) encodes glutamate dehydrogenase 1, which catalyzes the oxidative deamination of glutamate.

**Methods:**

We analyzed TCGA data using R language software and used multiple online databases to explore the relationship of GLUD1 with signaling pathways and drug sensitivity as well as GLUD1 protein expression and methylation.

**Results:**

The results showed that *GLUD1* mRNA expression was reduced in tumor tissues and correlated with the progression of ccRCC. Univariate and multivariate Cox analysis showed that GLUD1 could be used as a prognostic marker for ccRCC. GLUD1 expression in ccRCC was associated with immune cells infiltration and multiple classical signaling pathways. In addition, *GLUD1* mRNA expression was related to drug sensitivity.

**Conclusions:**

These findings provide new ideas for finding new prognostic molecular markers and therapeutic targets for ccRCC.

**Supplementary Information:**

The online version contains supplementary material available at 10.1186/s40001-024-01649-2.

## Introduction

Renal cell carcinoma (RCC) has varying incidence and mortality rates in different countries and is more common in developed countries [1]. According to the 2018 cancer data report, more than 400,000 new cases of RCC were reported worldwide, resulting in 175,000 deaths [2]. Although RCC occurs at all ages, most cases are first diagnosed between 40 and 60 years of age, and approximately 60% of RCC patients have insidious of initial symptoms, with more than 25% of patients having metastases by the time of diagnosis [3]. Smoking, age, hypertension and obesity are risk factors for RCC [4]. Radical or partial nephrectomy is the standard of care for early-stage disease, but innovative ablation techniques offer another treatment option for small renal lesions [5, 6]. However, surgery is not effective in treating patients with advanced or metastatic RCC. With the development of targeted drugs, the monoclonal antibodies ipilimumab and tremelimumab developed against the T cell immune checkpoints, have been used to treat patients with metastatic RCC patients [7, 8]. Some mTOR-targeting drugs such as everolimus and temsirolimus have also been approved for the treatment of metastatic RCC [9]. RCC is known as a metabolic disease, multiple genes involved in regulating the tumor are able to regulate various metabolic pathways such as glucose metabolism, amino acid metabolism and adenosine triphosphate [10–12]. The widespread metabolic reprogramming that occurs in RCC contributes to the poor prognosis and survival [13]. Glutamate Dehydrogenase 1 (GLUD1) encodes glutamate dehydrogenase 1, a mitochondrial matrix enzyme involved in regulating metabolic pathways, which may be a molecular marker with potential prognostic value.

Glutamate dehydrogenase 1 (GLUD1), also known as glutamate dehydrogenase 1 (GDH1), is the key enzyme in the conversion of glutamate to α-ketoglutarate (α-KG) during glutamine hydrolysis. A large number of studies have shown that GLUD1 is associated with hyperinsulinemia and neurological diseases, but its role in tumors has not been fully elucidated. Study suggests that high GLUD1 expression in breast cancer is associated with better prognosis and may predict response to chemotherapy in patients with triple-negative breast cancer [14]. In ccRCC, GDH1 is translocated from mitochondria to the cytoplasm and degraded following amino acid deprivation or mTORC1 inhibition, and ultimately leads to cellular preservation of nutrients for survival, suggesting that GDH1 plays an important role in the response of ccRCC to amino acid deficiency [15]. In addition, it has been shown that GLUD1 overexpression can inhibit ccRCC progression by suppressing PI3K/Akt/mTOR pathway activation [16]. In LKB1-deficient lung cancer, as a product of GDH1, α-KG can promote tumor metastasis via CamKK2-AMPK signaling [17]. These studies suggest that GLUD1 has different roles in tumors. In this study, we analyzed GLUD1 in ccRCC based on The Cancer Genome Atlas Program (TCGA) database to explore its value potential as a molecular marker of prognosis.

## Materials and methods

### TCGA database

The Cancer Genome Atlas (TCGA) is a large reference database for cancer research, storing a wide range of cancer-related histological data [18]. In this paper, we analyzed the expression of GLUD1 in ccRCC based on the TCGA database and predicted the relationship between GLUD1 and survival in ccRCC patients. The expression data files downloaded from TCGA were processed using R language software (version 4.2.3; https://www.rproject.org/) and the limma package. The *GLUD1* mRNA expression and clinical data were analyzed and visualized by GraphPad Prism 8. Immune cells infiltration was calculated by the cibersort algorithm and *p* < 0.05 was used as a screening value.

### UALCAN

The University of Alabama at Birmingham Cancer data analysis Portal (UALCAN) (http://ualcan.path.uab.edu) is a powerful database for online analysis of TCGA. In addition, UALCAN is able to analyze proteomics data from CPTAC and provides a variety of visualization capabilities such as differential protein expression, DNA methylation and survival analysis [19]. In this study, we used UALCAN to obtain the genes associated with GLUD1 in ccRCC and explored the expression of GLUD1 protein and mRNA expression in pan-cancer and ccRCC. Expression of GLUD1 in multiple signaling pathways was also explored in UALCAN.

### GEPIA2

Gene Expression Profiling Interactive Analysis (GEPIA2) (http://gepia2.cancer-pku.cn/#index) is a user-friendly interactive website with data derived from TCGA and GETx sequencing expression profiles. GEPIA2 is able to provide a variety of visualizations for gene differential expression, survival analysis, and other visualizations [20]. In this study, we referred GLUD1 to GEPIA2 to analyze its effect on the overall and disease-free survival of ccRCC patients.

### TIMER

Tumor Immune Estimation Resource (TIMER) (https://cistrome.shinyapps.io/timer/) is a website that can be used to comprehensively study the molecular characterization of tumor–immune interactions, offering six major analysis modules including gene expression [21]. In this study, we used TIMER to explore *GLUD1* mRNA expression in pan-cancer.

### SMART

Shiny Methylation Analysis Resource Tool (SMART) (http://www.bioinfo-zs.com/smartapp/) is a website application for comprehensive analysis of DNA methylation data from TCGA projects, providing visualization functions such as pan-cancer methylation mapping and differential methylation analysis [22]. We explored the methylation of GLUD1 in pan-cancer using SMART and analyzed in detail the methylation of GLUD1 detected by multiple methylation probes in ccRCC.

### GSCA

Gene Set Cancer Analysis (GSCA) (http://bioinfo.life.hust.edu.cn/GSCA) is a user-friendly web server that integrates expression, mutation and drug sensitivity for 33 cancer types [23]. In this study, we used GSCA to analyze and visualize the relationship between *GLUD1* mRNA expression and drug sensitivity based on Genomics of Drug Sensitivity in Cancer (GDSC) [24] and Cancer Therapeutics Response Portal (CTRP) database.

### Statistical analysis

Statistical analyses were performed using R (version 4.2.3) and Graphpad Prism 8.

All data in the online database are analyzed by their own statistical software. Differences of *p* < 0.05 were considered statistically significant.

## Results

### GLUD1 expression is decreased in ccRCC

**Fig. 1:**
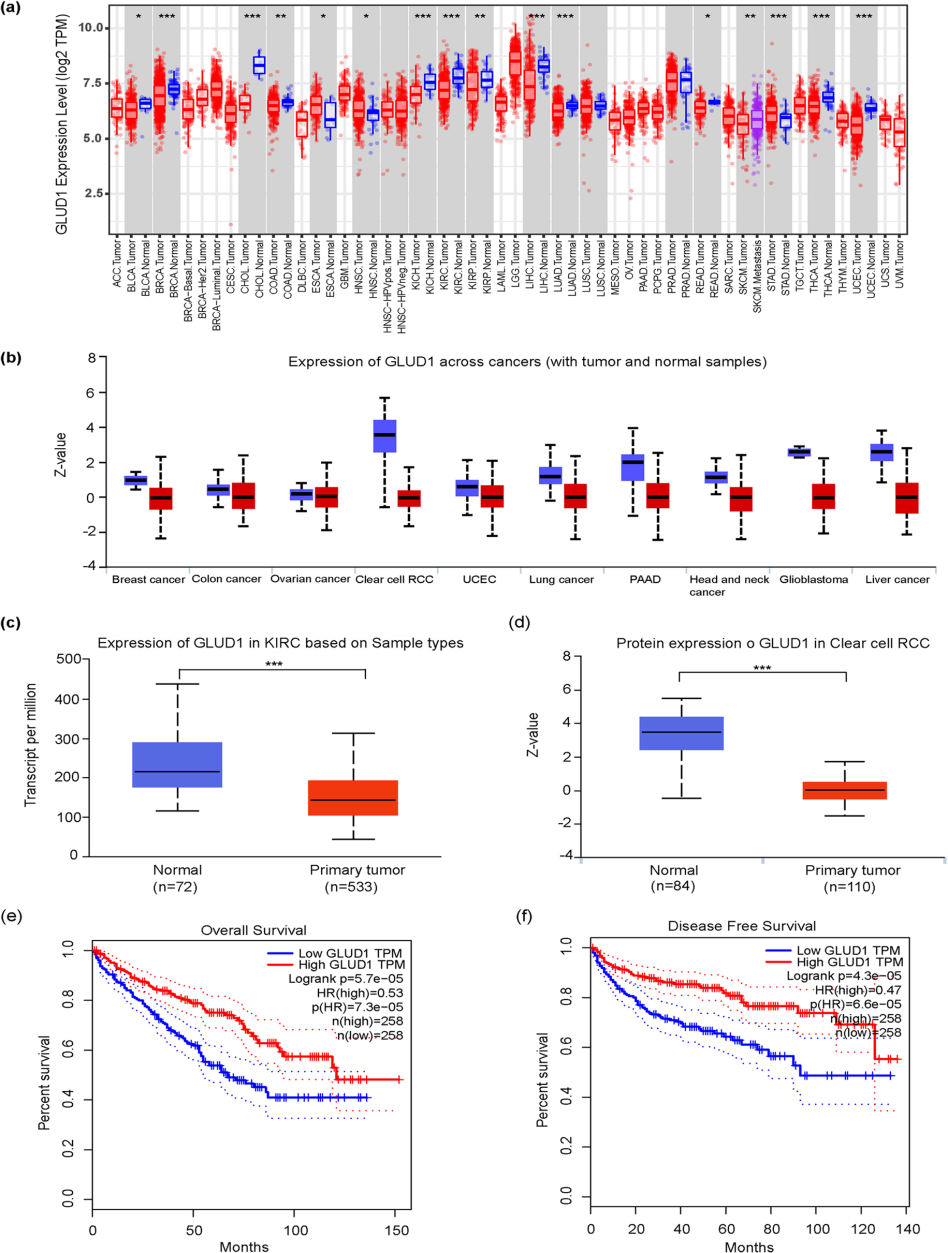
GLUD1 expression is reduced in ccRCC. **a** Expression levels of *GLUD1* mRNA in pan-cancer (https://cistrome.shinyapps.io/timer/) *, *p* < 0.05, **, *p* < 0.01, ***,*p* < 0.001. **b** Protein expression levels of GLUD1 in pan-cancer (http://ualcan.path.uab. edu). **c**, **d** Differences in expression of *GLUD1* mRNA and protein between normal and primary ccRCC tissues (http://ualcan.path.uab.edu) ***, *p* < 0.001. **e**, **f** Overall and disease-free survival of high and low GLUD1 expression groups in ccRCC patients (http://gepia2.cancer-pku.cn/#index)

It has been reported that RCC is a “metabolic disease”, mainly because a series of mutated genes are involved in the regulation of multiple metabolic pathways during the development of RCC [25]. GLUD1 encodes glutamate dehydrogenase 1 (GDH1), a key enzyme in glutamate metabolism, which is thought to be localized in the mitochondrial matrix. GDH1 catalyzes the oxidation of glutamate to produce α-KG, an intermediate involved in the tricarboxylic acid cycle. We submitted GLUD1 to TIMER to explore its expression in pan-cancer and the result showed that mRNA expression of GLUD1 was decreased in most tumors tissues compared with normal tissues (Fig. 1a), the result was generally consistent with the expression of GLUD1 protein in pan-cancer in CPTAC, and the difference in GLUD1 protein expression between normal and tumor tissues was more pronounced in ccRCC (Fig. 1b). We then explored GLUD1 expression in ccRCC separately in the UALCAN, and it was clear that GLUD1 mRNA (Fig. 1c) and protein (Fig. 1d) expression showed a decreasing trend in tumor tissues, and these results suggest that GLUD1 may be a suppressor factor in most tumors, including ccRCC. We obtained the effect of GLUD1 on the survival using the GEPIA2 website, and the results indicated that higher GLUD1 expression resulted in better survival outcomes both for overall survival (Fig. 1e) and disease-free survival (Fig. 1f) of ccRCC patients.

### GLUD1 expression is related to the progression and prognosis of ccRCC

**Fig. 2:**
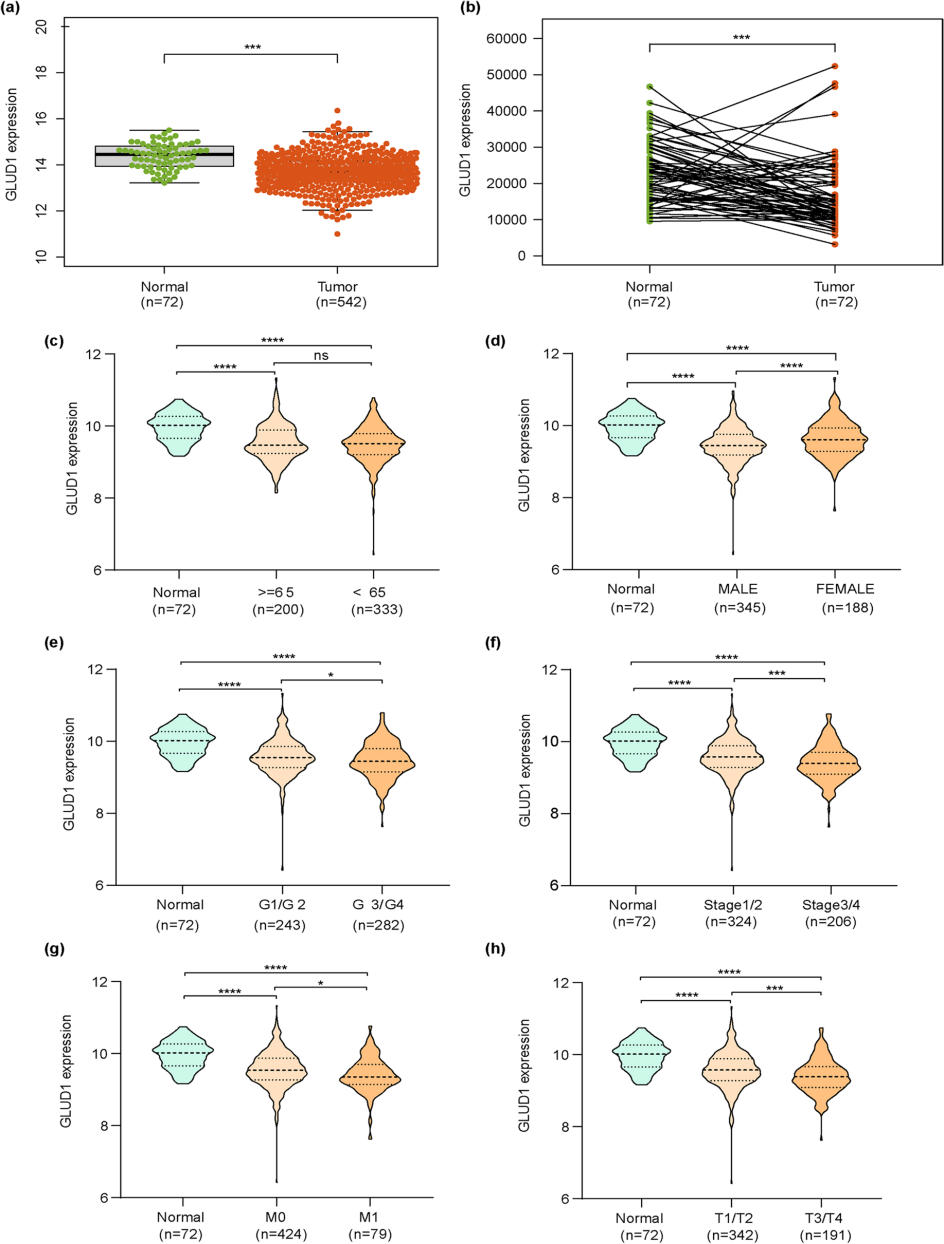
GLUD1 expression is correlated with ccRCC progression. **a** Differences in *GLUD1* mRNA expression in normal and tumor samples of TCGA,***, *p* < 0.001. **b** Expression of *GLUD1* mRNA in 72 groups of ccRCC and normal paired samples,***, *p* < 0.001. **c** Expression of *GLUD1* mRNA in ccRCC patients at different ages. **d** Expression of *GLUD1* mRNA in ccRCC patients by gender. **e** Differential expression of *GLUD1* mRNA in different grades of ccRCC. **f** Differential expression of *GLUD1* mRNA in different stages of ccRCC. **g** Differential expression of *GLUD1* mRNA in early and distantly metastasized ccRCC. **h** Differential expression of *GLUD1* mRNA in different tumor sizes

**Fig. 3:**
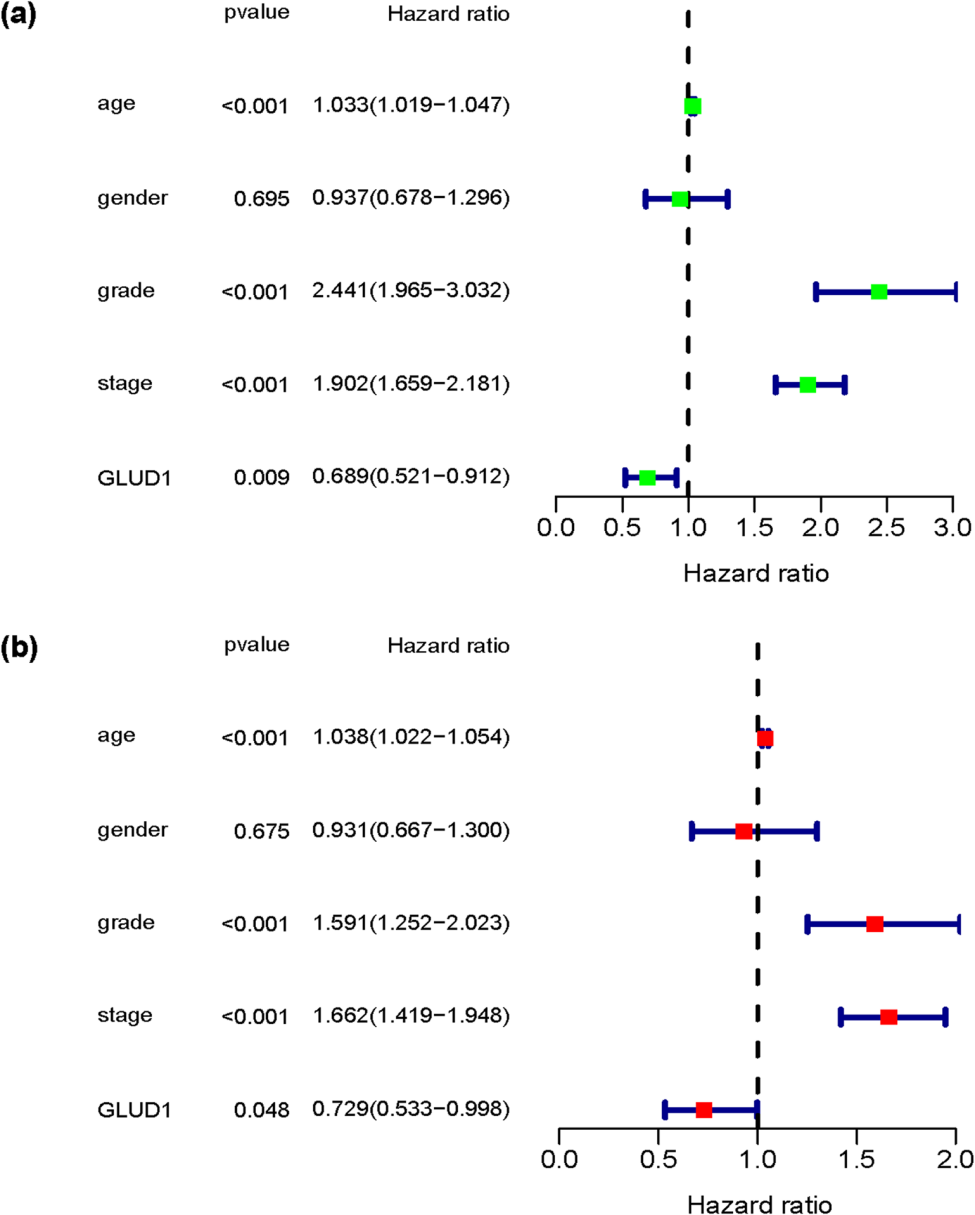
GLUD1 is an independent prognostic factor for ccRCC. **A** Univariate Cox analysis was used to validate the possibility of clinicopathological features and GLUD1 as a prognostic factor for ccRCC. **B** Multivariate Cox analysis was used to validate the possibility of GLUD1 as an independent prognostic marker for ccRCC

To further investigate the effect of GLUD1 on ccRCC, we downloaded the gene expression data of ccRCC from the TCGA database and analyzed the expression of GLUD1, as well as the clinical data of ccRCC patients. The results showed that the expression of GLUD1 in normal tissues was significantly higher than tumor tissues in unpaired samples (Fig. 2a), while the expression of GLUD1 in 72 paired samples showed the same results (Fig. 2b). By analyzing the association between GLUD1 and clinicopathological features of ccRCC, we found that GLUD1 expression was independent of age (Fig. 2c), but showed differences among genders (Fig. 2d). Moreover, GLUD1 showed a trend towards reduced expression in more malignant progression of ccRCC. In tumor grade, GLUD1 expression was lower in grade3/4 (G3/4) than in grade1/2 (G1/2) (Fig. 2e). This trend of reduced expression was also confirmed in stage (Fig. 2f), metastasis (M) (Fig. 2g) and tumor size (T) (Fig. 2h). These results may predict that GLUD1 is a suppressor factor in ccRCC. We performed univariate Cox analysis of the above clinical characteristics and GLUD1 expression with survival time and status of ccRCC patients, the results showed that GLUD1 was a protective factor for survival in ccRCC patients (Fig. 3a, Table 1), and multivariate Cox analysis exhibited the same results (Fig. 3b, Table 2), indicating that GLUD1 could be an independent factor for prognosis in ccRCC.

**Table 1:** Univariate Cox analysis

Characteristic	HR	HR.95L	HR.95H	*p*-value
Age	1.033	1.019	1.047	< 0.001
Gender	0.937	0.678	1.296	0.695
Grade	2.441	1.965	3.032	< 0.001
Stage	1.902	1.659	2.181	< 0.001
*GLUD1*	0.689	0.521	0.912	0.009

**Table 2:** Multivariate Cox analysis

Characteristic	HR	HR.95L	HR.95H	*p*-value
Age	1.038	1.022	1.054	< 0.001
Gender	0.931	0.667	1.300	0.675
Grade	1.591	1.252	2.023	< 0.001
Stage	1.662	1.419	1.948	< 0.001
*GLUD1*	0.729	0.533	0.998	0.048

### Low expression of GLUD1 is associated with methylation

**Fig. 4:**
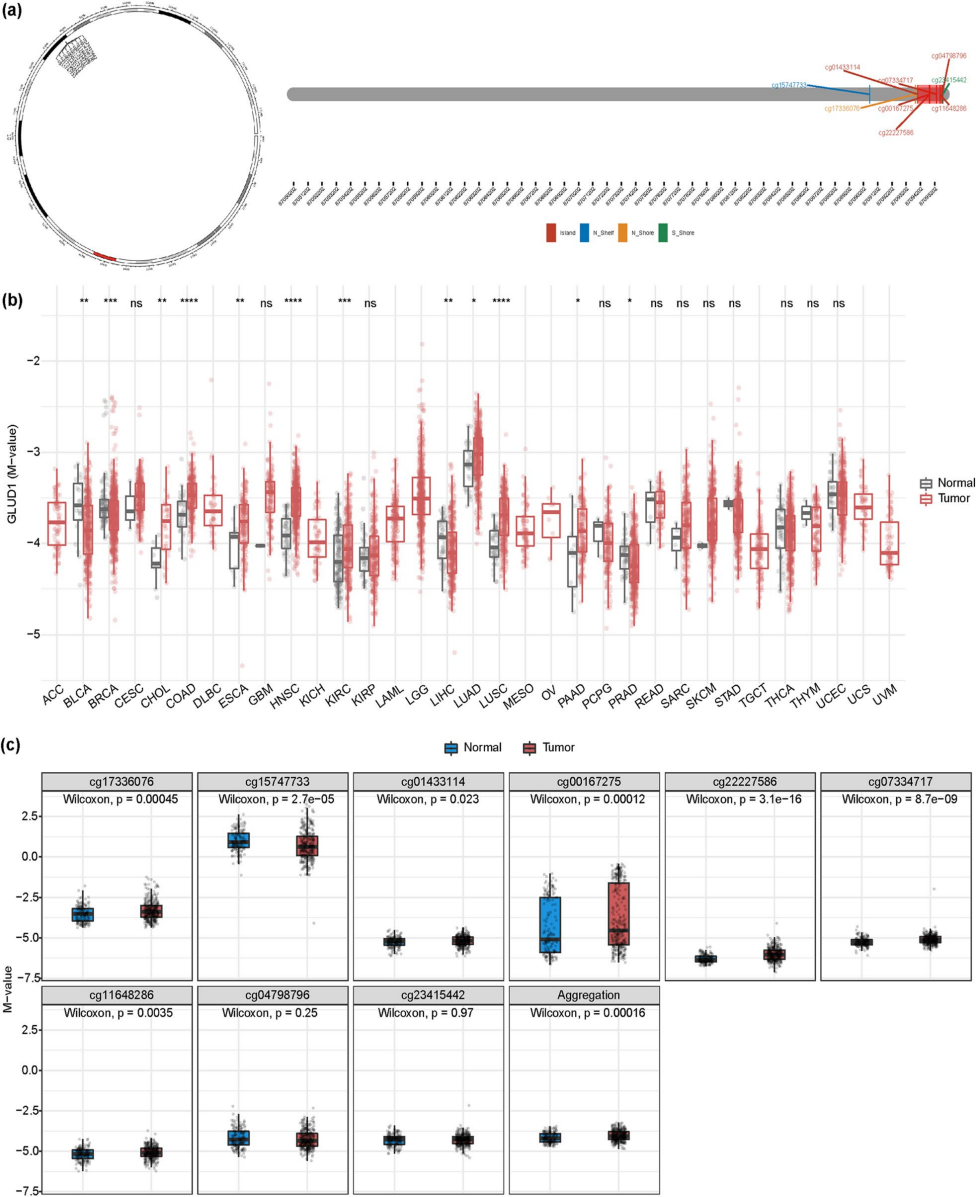
Low expression of GLUD1 in ccRCC is associated with methylation. **a** Information on different methylation probes designed for GLUD1. **b** Differential expression of GLUD1 methylation in pan-cancer,*, *p* < 0.05,**, *p* < 0.01,***, *p* < 0.001, *ns* no significance. **c** Methylation of GLUD1 in normal and tumor tissues of ccRCC detected by different probes

The above results indicated that GLUD1 expression was significantly reduced in RCC tissues compared with normal tissues. However, the cause of the reduced GLUD1 expression is not clear. We analyzed the methylation of GLUD1 through the SMART website. The information of different methylation probes designed for GLUD1 is shown in (Fig. 4a). The results revealed that the gene methylation of GLUD1 in pan-cancer. In most of the tumors, the methylation of GLUD1 was significantly elevated in tumor tissues compared to normal tissues (Fig. 4b). We next explored the methylation of GLUD1 detected by the above probes in ccRCC separately, except for the cg15747733 probe, most probes detected a high rate of GLUD1 methylation in tumor tissues of ccRCC (Fig. 4c). Gene methylation may be responsible for the reduced expression of GLUD1 in ccRCC, but gene expression is influenced by various factors such as transcription factors and non-coding RNA regulation, and there may be other reasons for reduced GLUD1 expression besides chromatin modification that still need to be explored in more depth.

### Expression of GLUD1 is associated with multiple signaling pathways

**Fig. 5:**
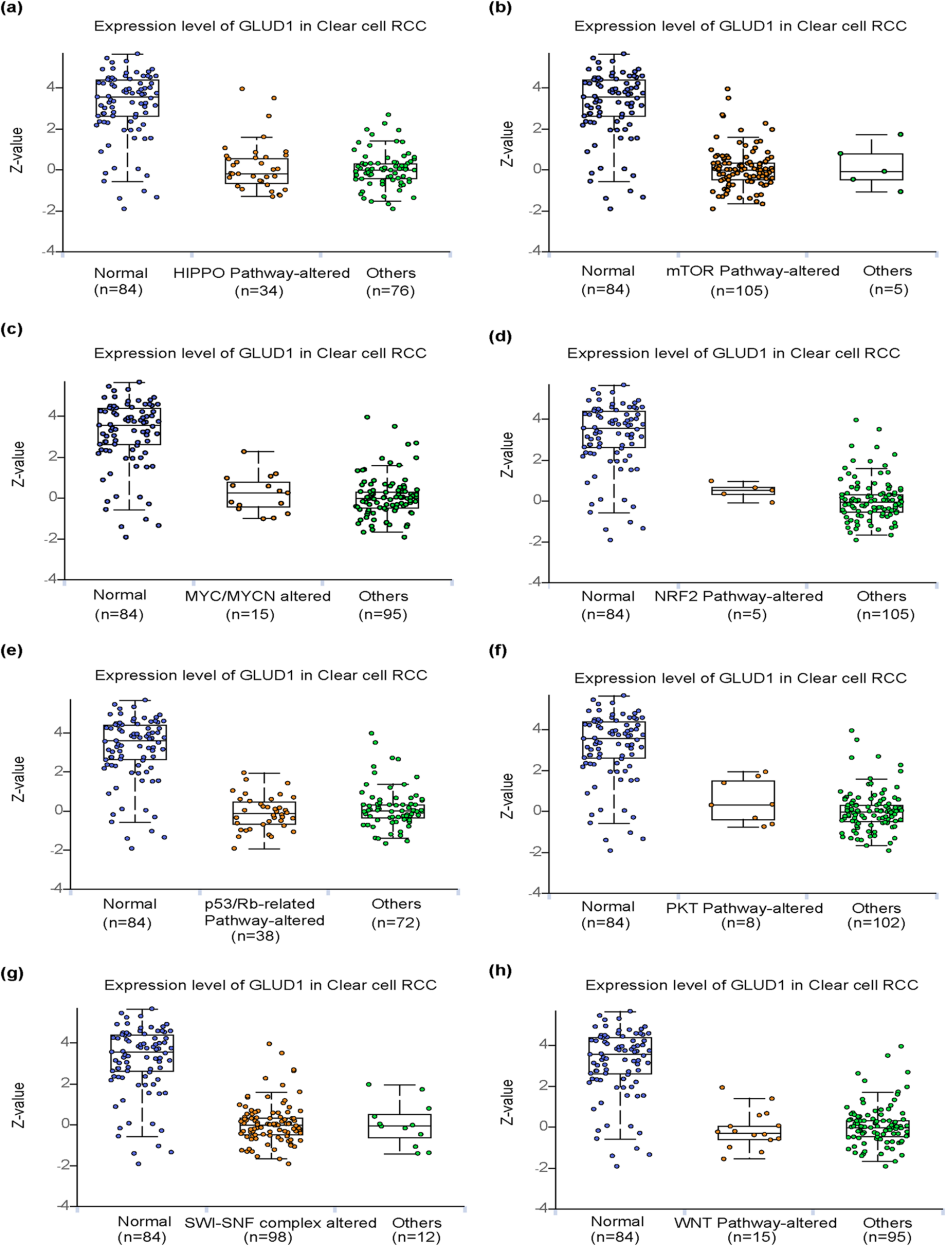
Expression of GLUD1 is associated with multiple pathways. **a** Differential protein expression of GLUD1 in the HIPPO pathway altered and normal tissues. **b** Differential protein expression of GLUD1 in the mTOR pathway altered and normal tissues. **c** Differential protein expression of GLUD1 in the MYC/MYCN altered and normal tissues. **d** Differential protein expression of GLUD1 in the NRF2 pathway altered and normal tissues. **e** Differential protein expression of GLUD1 in the p53/Rb-related pathway altered and normal tissues. **f** Differential protein expression of GLUD1 in the PKT pathway altered and normal tissues. **g** Differential protein expressionof GLUD1 in the  SWI/SNF complex altered and normal tissues. **h** Differential protein expression of GLUD1 in the  WNT pathway altered and normal tissues

Hippo pathway dysregulation is common in a variety of tumors and plays an important role in maintaining cell proliferation, reducing cell sensitivity to apoptosis, and regulating tumor metabolism [26]. Altered regulation of the PI3K–Akt–mTOR signaling pathway is one of the key pathways driving the malignant process, and they are involved in tumor development by controlling various cellular functions [27]. C-MYC, MYCN and MYCL are members of the MYC proto-oncogene family, and MYC is involved in the regulation of several processes including DNA damage repair, angiogenesis and matrix remodeling, dysregulation of MYC is common in a variety of tumors and is important in promoting tumorigenesis [28]. Studies have shown that aberrant activation of NRF2 in cancer can promote cell proliferation, the NRF2/KEAP1 signaling pathway is significant for the regulation of autophagy and the immune system, meanwhile, it is involved in tumorigenesis by regulating metabolic reprogramming [29]. Tumors are formed when normal cells lose control of growth and proliferate abnormally in response to various oncogenic factors. Deletion of the Rb family is associated with various aspects of tumorigenesis and affected the cell cycle as well as aging. Studies have shown that the simultaneous occurrence of oncogenic Ras and Rb family losses can strongly and consistently promote cell growth [30]. The Wnt/β-catenin signaling pathway is also known as the typical Wnt signaling pathway, and numerous studies have shown that dysregulation of the Wnt/β-catenin pathway is associated with the development and progression of a variety of malignancies [31]. Cancer genome sequencing studies have shown that SWI/SNF mutations, which increase cancer susceptibility and are associated with poorer prognosis in several cancers [32]. The above pathways basically cover the classical pathways found in the research of cancer development, and our study showed that the protein expression of GLUD1 was associated with these signaling pathways in ccRCC (Fig. 5a–h).

### Exploration of GLUD1 molecular functions

**Fig. 6:**
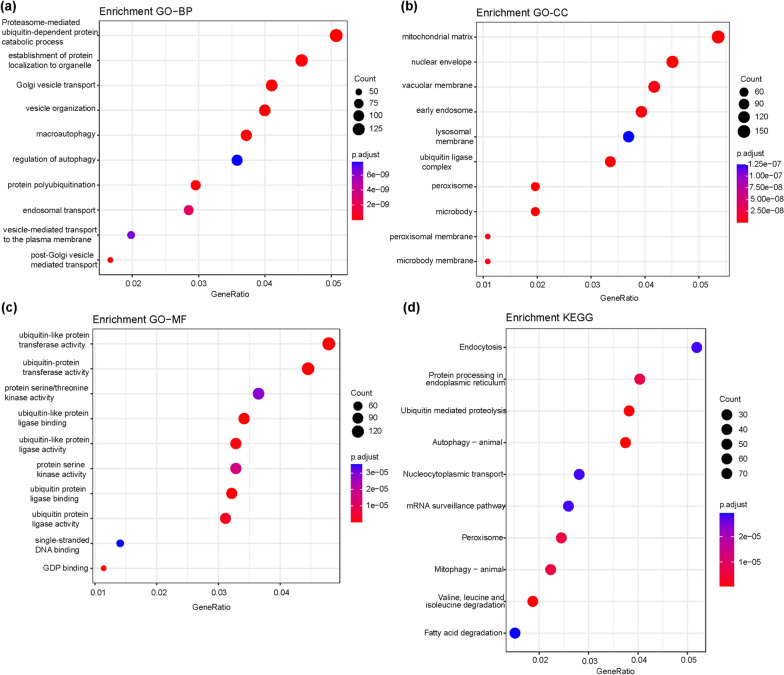
Pathway enrichment analysis of GLUD1-related genes. Genes related to GLUD1 in ccRCC were obtained from the UALCAN database and subjected to GO analysis and KEGG analysis. **a** GO analysis of biological processes. **b** GO analysis of cellular component. **c** GO analysis of molecular function. **d** KEGG analysis

For the enrichment of GLUD1 molecular functions in ccRCC, we downloaded the genes associated with GLUD1 using the UALCAN website and performed GO and KEGG analysis of these genes using R software. BP analysis suggested that these genes associated with GLUD1 in ccRCC were mainly enriched in proteasome-mediated ubiquitin-dependent protein catabolic processes, establishment of protein localization to organelle, Golgi vesicle transport, vesicle organization, macroautophagy, protein polyubiquitination and endosomal transport (Fig. 6a). The CC analysis predicted the cellular components of these related genes, including mitochondrial matrix, nuclear envelope, vacuolar membrane, early endosome, lysosomal membrane, ubiquitin ligase complexes peroxisome and microbody (Fig. 6b). MF analysis demonstrated the molecular functions of these related genes, and the result showed that these genes mainly have ubiquitin-like protein transferase activity, ubiquitin protein transferase activity, protein threonine/serine kinase activity, and ubiquitin-like protein ligase binding, ect (Fig. 6c). KEGG pathway analysis suggested molecular pathways in which these GLUD1-related genes were involved. The results showed that these pathways include endocytosis, protein processes in endoplasmic reticulum, ubiquitin-mediated proteolysis, autophagy, nucleocytoplasmic transport, mRNA surveillance pathway and peroxisome, etc. (Fig. 6d). These results revealed possible pathways for GLUD1 to perform its function in ccRCC.

### GLUD1 expression is correlated with immune cells infiltration

**Fig. 7:**
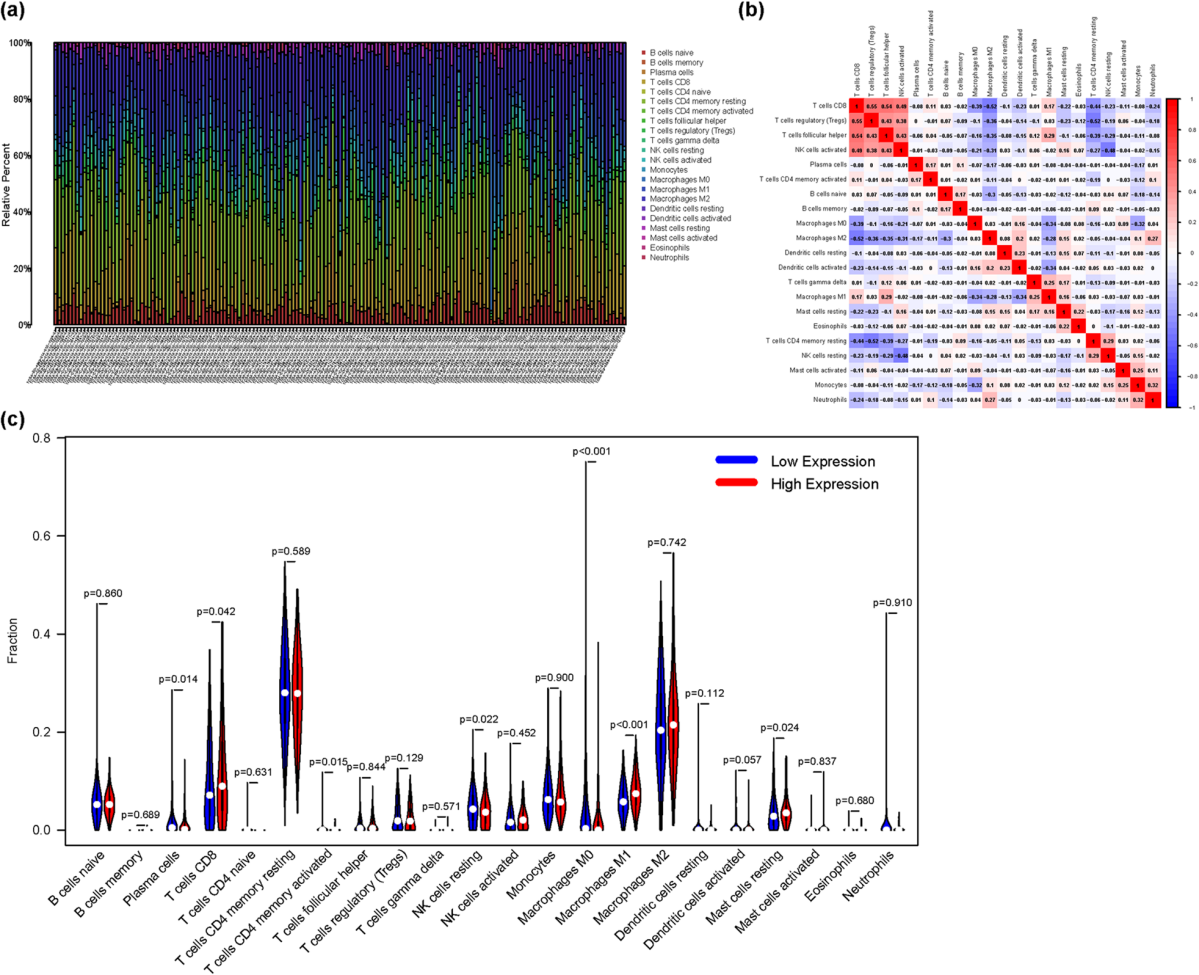
GLUD1 expression is associated with the tumor immune microenvironment of ccRCC. **a** Infiltration of various immune cells in TCGA samples. **b** Correlation between immune cells. **c** Differential expression of immune cells in the high and low expression groups of GLUD1

Tumors are complex diseases, and as research progresses, the tumor immune microenvironment, formed by the interaction of immune cells and cytokines, becomes a hot topic in research. More and more studies show that the immune system is significant in the progression and metastasis of tumors. In ccRCC, one of the key features of the immune microenvironment is the presence of multiple infiltrating immune cells, including T cells, B cells, natural killer cells and dendritic cells [33]. Our results demonstrated the expression of these immune cells in tumor samples of ccRCC (Fig. 7a), and the correlation between these immune cells (Fig. 7b). We divided GLUD1 expression into high and low groups by median and analyzed the differences in immune cells expression in the two groups, and the results indicated that multiple immune cells exhibited expression differences (Fig. 7c). These results suggested that GLUD1 expression and immune cells infiltration in ccRCC were correlated. Our study showed that GLUD1 was associated with the progression and prognosis of ccRCC and that immune cells infiltration may be one of the factors contributing to the outcome.

### GLUD1 expression is related to drug sensitivity

**Fig. 8:**
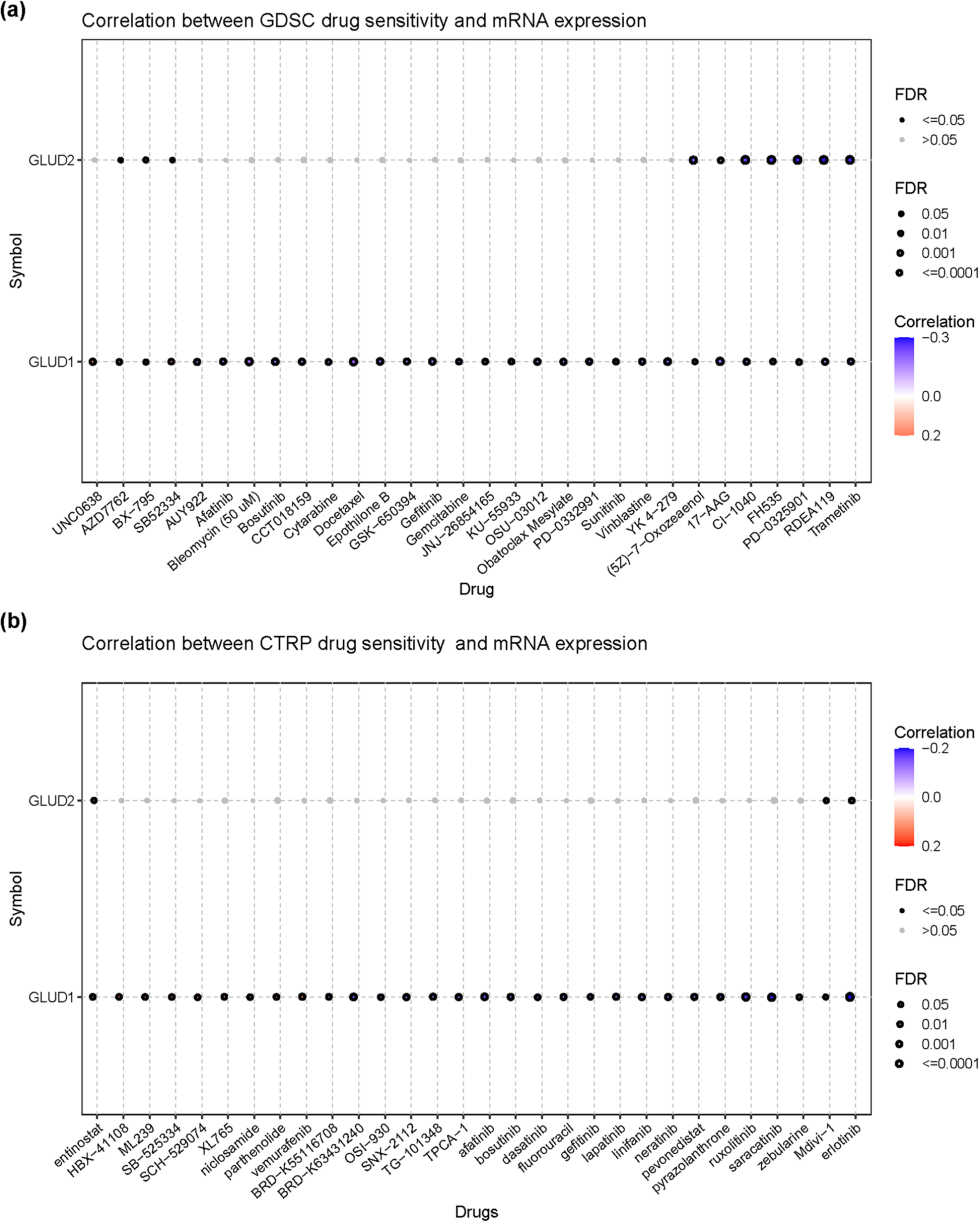
Relationship between *GLUD1* mRNA expression and drug sensitivity. **a** Relationship between *GLUD1* mRNA expression and drug sensitivity based on the GSDC database. **b** Relationship between *GLUD1* mRNA expression and drug sensitivity based on the CTRP database

Imaging and renal tissue biopsy have potential pitfalls in the differential diagnosis of renal disease, whereas radiogenomics has yielded promising results in determining stratified risk of disease, treatment options, follow-up strategies, and prognosis. However, its application in clinical practice has not yet been realized due to the limitations of retrospective studies and the small number of patients included in clinical trials [34]. The application of artificial intelligence and radiomics can improve the sensitivity, specificity, and accuracy of renal lesion detection and identification [35]. Although the development of radiomics may enable the goal of early detection and treatment of RCC, it is worth mentioning that approximately 20–30% of patients develop metastasis at an early stage of diagnosis, and nearly 40% of patients with localized RCC develop distant metastases after surgery [36]. For patients with advanced metastatic RCC, chemotherapy is the mainstay of treatment, but therapy for patients with advanced RCC remains a challenge due to individual differences in responsiveness and resistance. We analyzed the relationship between mRNA expression and drug sensitivity of two isoforms of the GLUD family through the GSCA database, the results showed that GLUD1 is correlated with multiple drug sensitivities (Fig. 8a, b, Additional file 2: Tables S1, S2). These results contribute to the understanding of new therapeutic hypotheses and support the future development of GLUD1-based chemotherapeutic agents.

## Discussion

Renal cell carcinoma accounts for 5 and 3% of all malignancies in men and women, respectively [37], and 30% of locally treated patients will experience recurrence and systemic progression in disease progression [38]. In this study, we analyzed clinical information and GLUD1 expression obtained from the TCGA database of ccRCC, results showed that GLUD1 expression was reduced in the tumor tissues and correlated with the malignancy of ccRCC, high expression of GLUD1 predicted a better prognosis for ccRCC patients. The results of the analysis suggested that GLUD1 could be an independent prognostic factor. Further exploration revealed that methylation may be responsible for the reduced expression of GLUD1 in tumors. The results of GLUD1 expression with multiple cancer-related pathways and the enrichment analysis of its related genes initially revealed the possible pathways of GLUD1 regulation in ccRCC. In addition, our results also revealed that GLUD1 expression was associated with immune cells infiltration and drug sensitivity in ccRCC.

GLUD1 encodes GHD1, which catalyzes the oxidative deamination of glutamate to produce α-KG and ammonia using NAD + and NADP + as cofactors [39]. There are two human glutamate dehydrogenase isoforms, GDH1 and GDH2, and it has been reported that they are mainly localized in mitochondria [40]. However, some researchers consider that they also localize to the endoplasmic reticulum [41]. Rosso et al. propose that GDH2 specifically targets mitochondria, while GDH1 is located in mitochondria and cytoplasm [42]. In breast cancer, the metabolic cycle of ammonia accelerates tumor proliferation. Ammonia accumulates in the tumor microenvironment and is directly used to generate amino acids to promote tumor growth through GDH activity [43]. In addition, α-KG is able to directly bind and activate IKKb and nuclear factor κB signaling in brain tumors, promoting glucose uptake and tumor cell survival through upregulation of GLUT1, and GDH1 S384 phosphorylation was associated with malignancy and prognosis of human glioblastoma [44].

GLUD1 acts differently in various tumors. Our study showed that GLUD1 was an inhibitor, low expression predicted more malignant of ccRCC and was associated with poor prognosis of ccRCC patients. Several studies have demonstrated that cancer progression is associated with multiple classical pathways, in our study, protein expression of GLUD1 was associated with alterations in those pathways compared to normal tissues. The results of enrichment analysis of GLUD1-related genes indicated that the molecular function of GLUD1 seems to be associated with ubiquitination. It has been previously described that in ccRCC, GDH1 translocates from the mitochondria to the cytoplasm following amino acid deprivation or mTORC1 inhibition, where it is ubiquitinated and degraded by E3 ligase [15]. We speculated whether GLUD1 might be differentially regulated in tumor and normal tissues, and through the GEPIA website we obtained GLUD1-related Top 100 genes in normal samples of ccRCC, we found that the molecular functions and pathways of these genes were mainly focused on metabolism-related aspects by GO and KEGG analysis (Additional file 1: Fig S1a–d). Whether the shift in GLUD1 function is related to its distribution of expression in cell mitochondria and cytoplasm under specific conditions needs to be further explored. Our study also suggested that GLUD1 expression was associated with immune cells infiltration in ccRCC. In conclusion, these results provided some theoretical basis for GLUD1 as a prognostic marker and therapeutic target for ccRCC, and suggested possible pathways in which GLUD1 may be involved as a way to affect ccRCC.

## Conclusion

In conclusion, our study demonstrated that GLUD1 is associated with the progression and prognosis of ccRCC. The protein expression of GLUD1 was altered in multiple signaling pathways, and immune cells infiltration showed differences in high and low GLUD1 expression groups. In addition, we found that *GLUD1* mNRA expression was correlated with multiple drug sensitivities. These results provide new insights into the search for new prognostic molecular markers and therapeutic targets for ccRCC.

### Supplementary Information

**Additional file 1: Fig S1.** Pathway enrichment analysis of GLUD1-related genes in normal samples of ccRCC. Genes related to GLUD1 in TCGA normal samples were obtained from the GEPIA database and subjected to GO analysis and KEGG analysis. (a) GO analysis of biological processes. (b) GO analysis of cellular component. (c) GO analysis of molecular function. (d) KEGG analysis.

**Additional file 2: Table S1.** Relationship between *GLUD1* mRNA expression and drug sensitivity based on GDSC database. **Table S2.** Relationship between *GLUD1* mRNA expression and drug sensitivity based on CTRP database.

## Data Availability

The datasets used and/or analyzed during the current study are available from the corresponding author on reasonable request.

## Abbreviations

GLUD1: Glutamate dehydrogenase 1
RCC: Renal cell carcinoma
ccRCC: Clear cell renal cell carcinoma
GDH1: Glutamate dehydrogenase 1
α-KG: α-Ketoglutarate
GEPIA: Gene Expression Profiling Interactive Analysis
UALCAN: The University of Alabama at Birmingham Cancer data analysis Portal
TCGA: The Cancer Genome Atlas
TIMER: Tumor Immune Estimation Resource
SMART: Shiny Methylation Analysis Resource Tool
GSCA: Gene Set Cancer Analysis
GDSC: Genomics of Drug Sensitivity in Cancer
CTRP: Cancer Therapeutics Response Portal
