# Supplementary figures and images for: Bioinformatics analysis identifies GLUD1 as a prognostic indicator for clear cell renal cell carcinoma
Source: Eur J Med Res. 2024 Jan 20;29:70. doi: 10.1186/s40001-024-01649-2 (PMC10799526; doi:10.1186/s40001-024-01649-2)

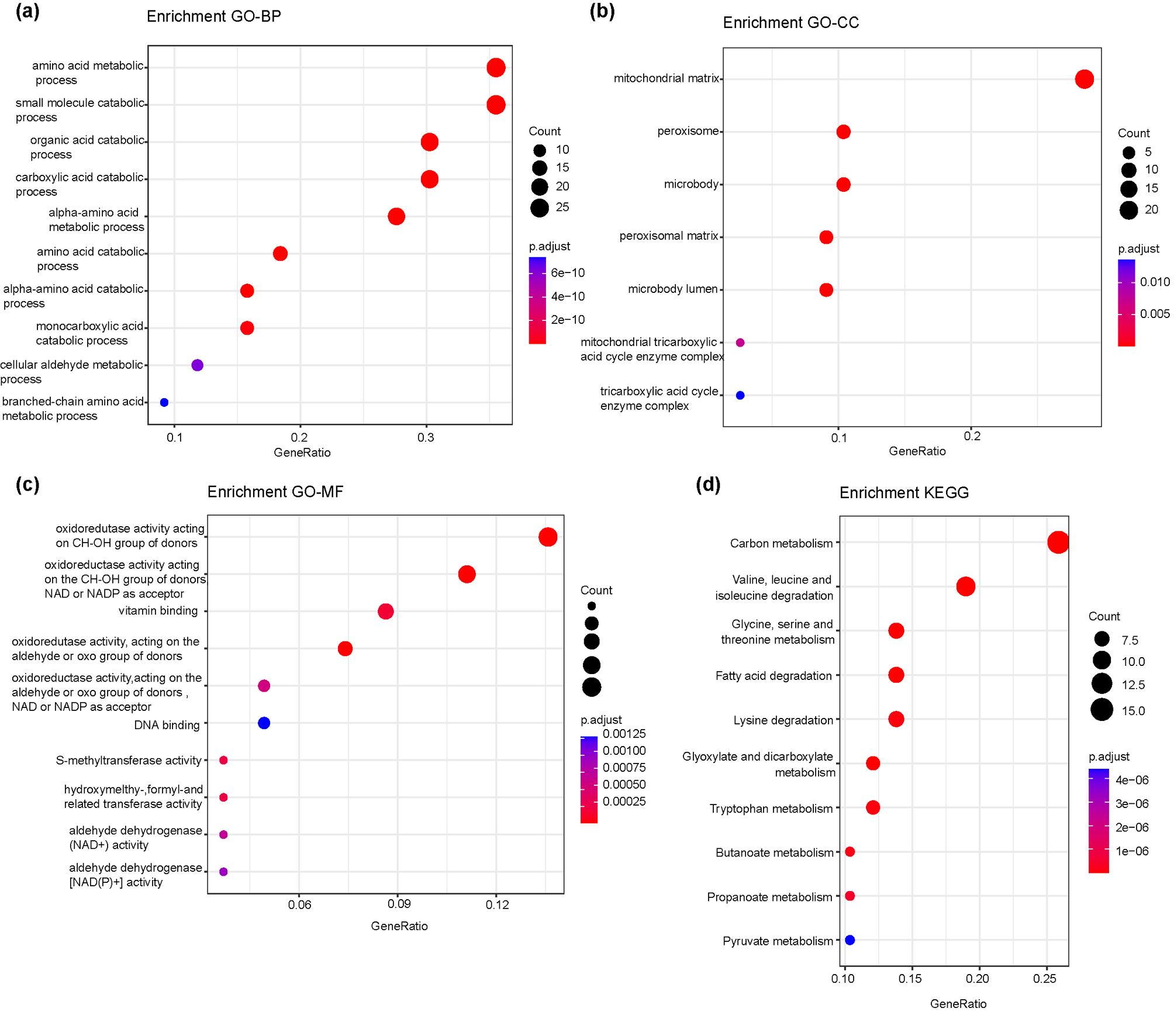

Supplement: Supplementary file 1 — Additional file 1: Fig S1. Pathway enrichment analysis of GLUD1-related genes in normal samples of ccRCC. Genes related to GLUD1 in TCGA normal samples were obtained from the GEPIA database and subjected to GO analysis and KEGG analysis. (a) GO analysis of biological processes. (b) GO analysis of cellular component. (c) GO analysis of molecular function. (d) KEGG analysis. [file 40001_2024_1649_MOESM1_ESM.tif]
