# Supplementary material for: Bioinformatics analysis identifies GLUD1 as a prognostic indicator for clear cell renal cell carcinoma
Source: Eur J Med Res. 2024 Jan 20;29:70. doi: 10.1186/s40001-024-01649-2 (PMC10799526; doi:10.1186/s40001-024-01649-2)
Supplement: Supplementary file 2 — Additional file 2: Table S1. Relationship between GLUD1 mRNA expression and drug sensitivity based on GDSC database. Table S2. Relationship between GLUD1 mRNA expression and drug sensitivity based on CTRP database. [file 40001_2024_1649_MOESM2_ESM.docx]

**Table S1 Relationship between *GLUD1* mRNA expression and drug sensitivity based on GDSC database**

| **symbol** | **drug** | **cor** | **fdr** |
| --- | --- | --- | --- |
| GLUD1 | (5Z)-7-Oxozeaenol | -0.120399315 | 0.001375981 |
| GLUD1 | 17-AAG | -0.194279017 | 6.0172E-08 |
| GLUD1 | 5-Fluorouracil | -0.077104853 | 0.03526194 |
| GLUD1 | 681640 | -0.116396973 | 0.030473027 |
| GLUD1 | A-770041 | -0.163827846 | 0.015150223 |
| GLUD1 | AICAR | -0.128484249 | 0.000746901 |
| GLUD1 | AKT inhibitor VIII | -0.098720102 | 0.018851856 |
| GLUD1 | AP-24534 | -0.094921497 | 0.015825097 |
| GLUD1 | AS601245 | -0.149483629 | 0.00054269 |
| GLUD1 | AUY922 | -0.18077297 | 8.80729E-06 |
| GLUD1 | AZD6482 | -0.09387077 | 0.021245556 |
| GLUD1 | AZD7762 | -0.138508393 | 0.000265514 |
| GLUD1 | Afatinib | -0.159091144 | 5.39138E-06 |
| GLUD1 | BEZ235 | -0.108497565 | 0.011235929 |
| GLUD1 | BX-795 | -0.121374719 | 0.002772396 |
| GLUD1 | Bexarotene | -0.110736304 | 0.030907167 |
| GLUD1 | Bleomycin | -0.150361835 | 0.000525407 |
| GLUD1 | Bleomycin (50 uM) | -0.195285269 | 2.00338E-08 |
| GLUD1 | Bosutinib | -0.202505042 | 7.36035E-07 |
| GLUD1 | CCT007093 | -0.087611853 | 0.03301245 |
| GLUD1 | CCT018159 | -0.178902266 | 0.00002195 |
| GLUD1 | CEP-701 | -0.099721208 | 0.010809369 |
| GLUD1 | CGP-60474 | -0.143695805 | 0.041116066 |
| GLUD1 | CI-1040 | -0.163761394 | 0.000023589 |
| GLUD1 | Cetuximab | -0.096004622 | 0.014363318 |
| GLUD1 | Cisplatin | -0.115605122 | 0.007894045 |
| GLUD1 | Cytarabine | -0.154535277 | 0.000208651 |
| GLUD1 | Dasatinib | -0.188211967 | 0.001757243 |
| GLUD1 | Docetaxel | -0.210354023 | 5.96633E-09 |
| GLUD1 | Doxorubicin | -0.110046423 | 0.032144192 |
| GLUD1 | EKB-569 | -0.088615307 | 0.021997829 |
| GLUD1 | Elesclomol | -0.115018949 | 0.004989908 |
| GLUD1 | Embelin | -0.139445135 | 0.001660855 |
| GLUD1 | Epothilone B | -0.203738267 | 2.24792E-06 |
| GLUD1 | Etoposide | -0.092684109 | 0.03036944 |
| GLUD1 | FH535 | -0.156320854 | 0.000120324 |
| GLUD1 | FTI-277 | -0.094203814 | 0.018709545 |
| GLUD1 | GDC0941 | -0.108859416 | 0.015299417 |
| GLUD1 | GSK-650394 | -0.189770981 | 0.00006856 |
| GLUD1 | GW843682X | -0.152010066 | 0.039272399 |
| GLUD1 | Gefitinib | -0.179118992 | 2.09139E-06 |
| GLUD1 | Gemcitabine | -0.161731476 | 0.000109447 |
| GLUD1 | HG-5-113-01 | -0.138643294 | 0.023221177 |
| GLUD1 | HG-6-64-1 | -0.148956076 | 0.000180786 |
| GLUD1 | JNJ-26854165 | -0.161528659 | 0.000111741 |
| GLUD1 | JNK Inhibitor VIII | -0.116154361 | 0.003827022 |
| GLUD1 | JNK-9L | -0.121226795 | 0.00805068 |
| GLUD1 | JQ12 | -0.096590649 | 0.044203327 |
| GLUD1 | JW-7-52-1 | -0.159091808 | 0.034283892 |
| GLUD1 | KU-55933 | -0.171999927 | 0.000129825 |
| GLUD1 | MLN4924 | -0.151097965 | 0.002152524 |
| GLUD1 | Midostaurin | -0.128821198 | 0.001359611 |
| GLUD1 | NPK76-II-72-1 | 0.097936725 | 0.005326404 |
| GLUD1 | NU-7441 | -0.158102473 | 0.003143396 |
| GLUD1 | Navitoclax | 0.107329039 | 0.00360304 |
| GLUD1 | OSU-03012 | -0.174054654 | 0.000031476 |
| GLUD1 | Obatoclax Mesylate | -0.170793978 | 0.000019962 |
| GLUD1 | PD-0325901 | -0.143438785 | 0.000147987 |
| GLUD1 | PD-0332991 | -0.193897156 | 4.25135E-06 |
| GLUD1 | PF-562271 | -0.159678614 | 0.000470452 |
| GLUD1 | Paclitaxel | -0.169830606 | 0.034092183 |
| GLUD1 | QL-X-138 | 0.073449617 | 0.046481153 |
| GLUD1 | RDEA119 | -0.147616451 | 0.000023649 |
| GLUD1 | RO-3306 | -0.133389793 | 0.000822533 |
| GLUD1 | SB 216763 | -0.112331754 | 0.01456839 |
| GLUD1 | SB52334 | 0.150981974 | 0.000092559 |
| GLUD1 | Saracatinib | -0.155593534 | 0.036536764 |
| GLUD1 | Shikonin | -0.097870826 | 0.034392807 |
| GLUD1 | Sunitinib | -0.234388996 | 0.000095319 |
| GLUD1 | TAE684 | -0.213535766 | 0.001848774 |
| GLUD1 | TL-2-105 | 0.082428035 | 0.025741605 |
| GLUD1 | TW 37 | -0.11198833 | 0.004969646 |
| GLUD1 | Temsirolimus | -0.104485906 | 0.01444582 |
| GLUD1 | Thapsigargin | -0.109624838 | 0.025721082 |
| GLUD1 | Tipifarnib | -0.145073312 | 0.001697587 |
| GLUD1 | Trametinib | -0.160120462 | 6.4196E-06 |
| GLUD1 | UNC0638 | 0.145007393 | 0.000022435 |
| GLUD1 | VX-11e | -0.087027104 | 0.043726301 |
| GLUD1 | Vinblastine | -0.159109233 | 0.000047136 |
| GLUD1 | Vinorelbine | -0.095299976 | 0.045993237 |
| GLUD1 | WH-4-023 | -0.140685456 | 0.038611387 |
| GLUD1 | WZ-1-84 | -0.153590371 | 0.02326768 |
| GLUD1 | WZ3105 | 0.075545428 | 0.035719719 |
| GLUD1 | XAV939 | -0.076863202 | 0.046640223 |
| GLUD1 | XMD13-2 | 0.074617201 | 0.039354958 |
| GLUD1 | YK 4-279 | -0.208146688 | 8.99152E-07 |
| GLUD1 | YM201636 | 0.105969669 | 0.003256863 |
| GLUD1 | Z-LLNle-CHO | -0.150819591 | 0.019009011 |
| GLUD1 | piperlongumine | -0.123444945 | 0.00150105 |
| GLUD1 | selumetinib | -0.075479473 | 0.041237184 |

**Table S2 Relationship between *GLUD1* mRNA expression and drug sensitivity based on CTRP database**

| **symbol** | **drug** | **cor** | **fdr** |
| --- | --- | --- | --- |
| GLUD1 | ABT-737 | 0.098299629 | 0.023933429 |
| GLUD1 | AZ-3146 | -0.089721407 | 0.030886634 |
| GLUD1 | AZD7762 | -0.097955029 | 0.011360305 |
| GLUD1 | BRD-K45681478 | 0.087384741 | 0.046139734 |
| GLUD1 | BRD-K51490254 | 0.086134996 | 0.041233341 |
| GLUD1 | BRD-K55116708 | -0.108939322 | 0.011522833 |
| GLUD1 | BRD-K63431240 | -0.156318824 | 0.000107234 |
| GLUD1 | BRD6340 | -0.079883611 | 0.046424754 |
| GLUD1 | Compound 23 citrate | -0.089703629 | 0.023795501 |
| GLUD1 | DBeQ | 0.091317907 | 0.033552018 |
| GLUD1 | HBX-41108 | 0.149636137 | 0.003335952 |
| GLUD1 | KU 0060648 | -0.094817768 | 0.018906204 |
| GLUD1 | KU-60019 | -0.087237537 | 0.027787741 |
| GLUD1 | MK-1775 | -0.088119535 | 0.026011948 |
| GLUD1 | ML239 | 0.12306077 | 0.002500036 |
| GLUD1 | MST-312 | 0.079162594 | 0.04346065 |
| GLUD1 | Mdivi-1 | -0.100460644 | 0.015164351 |
| GLUD1 | N9-isopropylolomoucine | -0.084674147 | 0.039008175 |
| GLUD1 | NVP-TAE684 | -0.089997212 | 0.045904947 |
| GLUD1 | OSI-930 | -0.119629649 | 0.006005978 |
| GLUD1 | PF-573228 | -0.087305681 | 0.02862887 |
| GLUD1 | PI-103 | 0.084611037 | 0.041039012 |
| GLUD1 | SB-525334 | 0.119858436 | 0.008821902 |
| GLUD1 | SCH-529074 | 0.125426211 | 0.004585384 |
| GLUD1 | SNX-2112 | -0.125609883 | 0.0007904 |
| GLUD1 | TG-101348 | -0.120224251 | 0.002397726 |
| GLUD1 | TPCA-1 | -0.129288799 | 0.000830873 |
| GLUD1 | XL765 | 0.124852509 | 0.012888147 |
| GLUD1 | afatinib | -0.159197065 | 0.000191215 |
| GLUD1 | bleomycin A2 | -0.099150069 | 0.046720195 |
| GLUD1 | bosutinib | -0.14306501 | 0.000335529 |
| GLUD1 | canertinib | -0.099409105 | 0.024171238 |
| GLUD1 | crizotinib | -0.082560799 | 0.037286856 |
| GLUD1 | dacarbazine | -0.090870448 | 0.020357748 |
| GLUD1 | dasatinib | -0.121481481 | 0.004202075 |
| GLUD1 | decitabine | -0.094525941 | 0.013022362 |
| GLUD1 | entinostat | 0.11481279 | 0.002963127 |
| GLUD1 | erlotinib | -0.198179437 | 5.22893E-07 |
| GLUD1 | fluorouracil | -0.124190717 | 0.001228074 |
| GLUD1 | gefitinib | -0.113086776 | 0.012258955 |
| GLUD1 | ibrutinib | -0.110518728 | 0.041321516 |
| GLUD1 | lapatinib | -0.128923953 | 0.002213463 |
| GLUD1 | linifanib | -0.12663597 | 0.000983736 |
| GLUD1 | neratinib | -0.133823716 | 0.001046007 |
| GLUD1 | niclosamide | 0.112816303 | 0.00677235 |
| GLUD1 | parthenolide | 0.132037979 | 0.009216385 |
| GLUD1 | pevonedistat | -0.124901503 | 0.001511964 |
| GLUD1 | pyrazolanthrone | -0.143343199 | 0.001230365 |
| GLUD1 | ruxolitinib | -0.175950298 | 8.20329E-06 |
| GLUD1 | saracatinib | -0.191545669 | 2.53229E-06 |
| GLUD1 | tipifarnib-P2 | -0.111555208 | 0.044159305 |
| GLUD1 | vemurafenib | 0.146307305 | 0.002356435 |
| GLUD1 | zebularine | -0.108758473 | 0.005765416 |
